# Renal Denervation After USA FDA Approval: An Update from an Interventional Cardiologist’s Perspective

**DOI:** 10.3390/jcm14103554

**Published:** 2025-05-19

**Authors:** Jiandong Zhang, Peter M. Belford, George A. Stouffer

**Affiliations:** 1 Department of Cardiovascular Medicine, Wake Forest University School of Medicine, Winston-Salem, NC 27157, USA; belford@wakehealth.edu; 2 Division of Cardiology, Department of Medicine, University of North Carolina, Chapel Hill, NC 27514, USA; rick_stouffer@med.unc.edu; 3 Department of Medicine, McAllister Heart Institute, University of North Carolina, Chapel Hill, NC 27514, USA

**Keywords:** renal denervation, hypertension, sympathetic nervous system, resistant hypertension

## Abstract

In late 2023, the U.S. Food and Drug Administration (FDA) approved two renal denervation (RDN) systems for the treatment of hypertension. Several professional societies, including the Society of Cardiovascular Angiography and Intervention (SCAI), the American Heart Association (AHA), and numerous European associations, have recognized the potential role of RDN in managing hypertension. Despite widespread enthusiasm from clinicians, patients, and the industry, the American Medical Association’s Current Procedural Terminology (CPT) panel rejected the introduction of new codes for renal denervation at its September 2024 meeting. This article analyzes the latest evidence from clinical trials and registries, reviews current challenges in clinical practice, and explores the role of contemporary hypertension treatment from the perspective of interventional cardiologists.

## 1. The Burden of Hypertension and Inadequate Control

Hypertension remains a significant global health issue, affecting over 1.3 billion people worldwide [1]. It is a leading risk factor for cardiovascular diseases, stroke, chronic kidney disease, and premature mortality. Despite the availability of numerous pharmacological treatments and lifestyle interventions, a large proportion of hypertensive patients fail to achieve adequate blood pressure (BP) control. Data from the National Health and Nutrition Examination Survey (NHANES) reveals that only 51.1% of adult hypertensive patients achieved controlled hypertension between August 2021 and August 2023 [2]. Several factors contribute to this gap, including poor medication adherence and comorbid conditions, highlighting the urgent need for more effective and innovative therapeutic strategies. Percutaneous RDN has emerged as a promising adjunctive therapy, particularly for patients with resistant hypertension, offering another therapeutic option for this pressing public health issue.

## 2. What Is Renal Denervation?

Modulating the sympathetic nervous system (SNS) is a well-established strategy for hypertension management [3]. Surgical sympathectomy, first introduced in the 1930s, demonstrated durable blood pressure-lowering effects in severe and malignant hypertension. However, it was associated with high mortality rates (up to 10%) and severe complications, such as postural hypotension and abdominal paralysis [4]. The development of sympathetic-blocking drugs led to the abandonment of surgical sympathectomy.

Recent advancements in our understanding of the pathophysiology of hypertension have reaffirmed the SNS’s pivotal role in blood pressure regulation, particularly through the kidneys. The kidneys both generate and receive sympathetic signals. Afferent fibers contribute to chronic sympathetic overactivity, while efferent fibers originating in the central nervous system drive vasoconstriction, sodium retention, and the activation of the renin–angiotensin–aldosterone system, culminating in sustained hypertension and end-organ damage. These insights have fueled the development of RDN. The advent of transcatheter techniques has since facilitated the development of percutaneous approaches.

RDN involves disrupting the renal nerves to mitigate sympathetic overdrive. Initially, surgical methods such as transection, electrocautery, cryoablation, and thermal or chemical ablation were used. The advancements of transcatheter techniques have since facilitated the development of percutaneous approaches.

Over the past few decades, significant progress has been made in refining catheter-based RDN. Three primary technologies have been extensively studied (see Table 1). Regardless of the RDN method, pre-procedural imaging is essential for evaluating renal and adrenal anatomy, excluding secondary causes of hypertension, and identifying anatomical contraindications. Aortography and selective renal artery angiography help locate renal artery origins and confirm eligibility. Post-treatment angiography ensures the absence of renal artery injuries. Multidisciplinary follow-up is advised to optimize patient outcomes.

## 3. Latest Evidence from Clinical Trials and Registries

The journey of catheter-based RDN has been marked by both challenges and advancements. Initial enthusiasm was dampened by the results of the SYMPLICITY HTN-3 trial, which showed no significant difference in blood pressure reduction between the treatment and sham groups [5]. However, the field quickly adapted, with vigorous improvements in study designs, methodologies, and device technology [6] (see Table 2). A growing body of evidence now supports the safety and efficacy of transcatheter RDN, and several professional societies have incorporated RDN into their hypertension management guidelines [7].

A significant breaking point was reached in November 2023, when the FDA approved the first RDN systems: the Paradise Ultrasound RDN System (Recor Medical) [7] and the Symplicity Spyral Radiofrequency RDN System (Medtronic) [8]. These approvals underscore the growing recognition of RDN as a viable therapeutic option for hypertension management (see Table 3). Since then, new trials exploring various device systems for RDN continue to emerge. Below is a summary of noteworthy findings from recent studies.

In the SPYRAL HTN-ON MED pivotal trial, Kandzari et al. reported at TCT 2024 that RDN using the Simplicity device (Medtronic) was both safe and effective, leading to clinically meaningful and significant blood pressure reductions. After 24 h ambulatory blood pressure monitoring (ABPM), the RDN group saw a reduction of −12.1 mmHg compared to −7.0 mmHg in the sham group, with a treatment difference of −5.7 mmHg (*p* = 0.039) at 24 months [9]. This represents a notable improvement compared to earlier results at the 6-month benchmark, where no significant difference was seen between the two groups (−6.5 mmHg for RDN vs. −4.5 mmHg for sham; *p* = 0.12) [10]. This result aligns with the progressive reduction in blood pressure from the initial SPYRAL HTN-ON MED pilot trial with treatment differences of −7.4 mmHg at 6 months [11] and −11.2 mmHg at 24 months [12].

Similarly, data from the RADIANCE-HTN TRIO trial demonstrated the safety and durability of ultrasound-based RDN, with a consistent reduction in systolic blood pressure of −8 mmHg from 2 months to 36 months [13]. These results, alongside the promising outcomes from other RDN devices, support the medium-term efficacy of RDN, especially in patients with resistant hypertension despite multiple medications. The additional blood pressure reduction observed in these studies raises intriguing possibilities for mechanisms beyond sympathoinhibition, such as metabolic remodeling.

In a novel approach, Kandzari et al. also reported promising results using a microneedle system (Peregrine), which delivered dehydrated alcohol for RDN. This single treatment led to a modest but significant reduction in 24 h ambulatory systolic blood pressure, with a treatment difference of −3.2 mmHg (*p* = 0.0487) in hypertensive patients on two to five medications [14]. Another study evaluating a basket-shaped, six-monopolar electrode radiofrequency system (Netrod™ RDN System), reported more substantial blood pressure reduction, with a treatment difference of −9.7 mmHg (*p* < 0.01) in Chinese hypertensive patients on a fixed-dose regimen of two medications [15]. Similarly, a recent *Circulation* publication on a 6F-based radiofrequency system reported comparable results, with a treatment difference of −9.4 mmHg (*p* < 0.001), and highlighted the potential for compatibility with radial access [16].

Moreover, a pilot study using a 4F, one-size-fits-all ultrasound-based RDN system (TIVUS, Sonivie, Rehovot, Israel) showed promising results, with an average reduction of 12 mmHg in daytime ambulatory systolic blood pressure over three months. Notably, 78.4% of patients were considered responders [17]. Following this, the THRIVE study, a randomized, double-blind, sham-controlled trial, received FDA approval to further evaluate this system.

In addition, both Medtronic and Recor are conducting post-FDA approval registries to gather real-world data on the long-term safety and effectiveness of their respective RDN systems [18,19].

## 4. Current Challenges in Clinical Practice of Renal Denervation

Expert consensus statements from Europe, Asia, and North America are in general agreement in support of RDN as a potential adjunct treatment in patients with uncontrolled hypertension despite maximizing the efforts at lifestyle modification and medication interventions, offering guidance on patient selection, shared decision making, procedural training, and post-procedure follow-up [20,21,22,23,24,25,26,27] (summarized in Table 3). As of 2025, regulatory approval has been granted in more than 60 countries, including the United States, marking a critical step towards its clinical implementation. However, significant challenges remain in translating regulatory clearance into widespread clinical adoption.

One of the foremost barriers to the widespread adoption of RDN is the inherent complexity of hypertension as a disease [28]. Hypertension is a multifactorial condition influenced by genetic predisposition, environmental factors, lifestyle choices, and behavioral habits. Standardized treatment approaches are difficult because many patients with uncontrolled hypertension are already on multiple antihypertensive medications. However, factors such as non-adherence, secondary causes of hypertension, and white-coat hypertension can complicate the assessment of true treatment resistance.

Patient selection remains an evolving challenge. While certain subgroups appear to derive greater benefit from RDN, ongoing research seeks to further refine which patient populations are ideal candidates [29]. Additionally, the long-term durability of RDN remains a concern, as the benefits of the procedure could be offset by medication non-adherence or poor lifestyle choices [30]. To address these issues, a multidisciplinary team approach involving cardiologists, nephrologists, hypertension specialists, and interventionalists is essential to ensuring appropriate patient selection and long-term management. Further details will be provided in the section of Topics of Interest.

Optimal procedural planning and techniques are critical for achieving safe and effective blood pressure reductions with catheter-based RDN therapy. Several factors need to be carefully addressed and even standardized to establish a successful RDN program:

- Preprocedural Imaging: Noninvasive imaging should be performed to rule out structural causes of secondary hypertension and to assess renal artery anatomy, including the presence of accessory arteries that may impact procedural efficacy.
- Contrast-Induced Nephropathy (CIN) Risk: As RDN relies on contrast-based imaging, proper hydration strategies and judicious contrast use are essential, particularly in patients with preexisting renal impairment.
- Pain Management: Due to the nonselective nature of nerve ablation, adequate analgesia and sedation are necessary to manage procedural pain and improve patient comfort.
- Anticoagulation and Antiplatelet Management: Unfractionated heparin should be administered during the procedure to maintain an activated clotting time (ACT) > 250 s. Additionally, aspirin loading followed by low-dose aspirin for one month post-procedure is recommended to minimize thrombotic risks.

While RDN has a highly favorable safety profile, proficiency in managing potential complications such as renal artery perforation, dissection, and vascular access complications are essential for a successful RDN program. As the procedure involves complex renal artery and nerve anatomy, operator expertise is critical to achieving consistent and safe outcomes [31]. Standardized training programs, procedural simulations, and hands-on experience with experienced operators can help mitigate variability in outcomes and ensure that RDN is performed safely and effectively across different healthcare settings.

One of the most significant barriers to the widespread clinical adoption of RDN, particularly in the United States, is the lack of reimbursement. In September 2024, the American Medical Association’s (AMA) CPT Editorial Panel rejected two proposed Current Procedural Terminology (CPT) codes for RDN, delaying potential reimbursement until at least 2025 [32]. However, organizations such as the American College of Radiology (ACR) have petitioned for CPT code revisions, including the addition of sympathetic denervation of renal arteries.

This delay has placed financial strain on hospitals and providers performing RDN procedures, as there is no formal reimbursement pathway despite growing clinical demand. Medicare’s New Technology Add-on Payment (NTAP) approval, which became effective 1 October 2024, offers temporary relief under the Medicare Hospital Inpatient Prospective Payment System (IPPS) [33]. Nevertheless, additional research is needed to demonstrate the long-term cost-effectiveness of RDN in reducing healthcare expenditures related to hypertension.

Moreover, most guidelines and recommendations list advanced chronic kidney disease (CKD) as either contraindications or an area of uncertainty for RDN. For instance, the European Society of Hypertension (ESH) recommends selecting patients with an estimated glomerular filtration rate (eGFR) above 40 mL/min/1.73 m^2^. This caution primarily stems from the fact that clinical trials to date have largely excluded advanced CKD patients, leading to a lack of robust efficacy and safety data in this subgroup. However, advanced CKD patients are known to exhibit heightened sympathetic activity, often accompanied by uncontrolled BP –features that theoretically make them ideal candidates for RDN. With ongoing research and emerging clinical data, it is reasonable to project that the indications for RDN may eventually expand to include patients with advanced CKD, pending further evidence on safety, efficacy, and long-term clinical outcomes [34].

## 5. Topics of Interest

Following FDA approval of two major RDN systems, the focus in the field has shifted beyond efficacy and safety to explore clinical long-term durability, patient selection, and broader cardiovascular benefits. These emerging topics will play a pivotal role in determining RDN’s place in routine clinical practice and its potential to reshape hypertension management.

The durability of RDN therapy and the potential for nerve regrowth remains a clinical focus in understanding its long-term efficacy. Clinical trials and real-world studies have demonstrated that the blood pressure-lowering effects of RDN can persist for over three years, with some studies showing a progressive reduction in blood pressure over time, as mentioned above [5,25]. Moreover, further BP reduction in patients undergoing RDN was observed with either a decreased or similar antihypertensive medication burden compared to baseline conditions. This was further supported in Global SYMPLICITY Registry DEFINE, which showed persistent SBP reductions from baseline, alongside a decrease in the number of antihypertensive medications 4.6 ± 1.4 at baseline to 4.3 ± 1.5 at 36 months (*p* < 0.001) [35].

Collectively, these findings suggest that RDN may induce structural and functional changes beyond immediate sympathetic nerve modulation. However, concerns regarding renal nerve regrowth have been raised, as previous studies have indicated that partial nerve regeneration can occur within months following denervation [36]. The clinical relevance of this phenomenon in humans remains uncertain [37,38]. This has prompted studies into optimizing ablation techniques, such as using more extensive or deeper energy delivery, to ensure more permanent disruption of sympathetic nerves.

As we discussed above, patient selection plays a crucial role in determining the efficacy of renal denervation but remains one of the most pressing unmet clinical needs. Substantial efforts have been devoted to identifying the responders and characterizing their key distinct clinical features. The foremost challenge is to define what constitutes a “responder”. Typically, responders are identified as subjects achieving a predetermined threshold of office systolic BP reduction (for example 5 or 10 mmHg). However, inherent visit-to-visit BP variability introduces substantial uncertainties into this definition, especially when it relies on single-point measurements. Additionally, any medication changes, and even the timing of administration (medications on the day of BP measurements) can significantly affect outcomes. Consequently, defining treatment responders requires a multifaced approach that incorporates variables such as the methods of BP measurement (ABPM, office BP, home BP), visit-to-visit variability, drug changes, etc.

Using data from multiple clinical trials, investigators have identified significant blood pressure reductions in select populations with unique clinical signatures [39]. Several markers associated with increased basal sympathetic activity such as heart rate [40], plasma renin activity [41], and arterial stiffness [42] have been proposed as potential predictors of procedural response. In contrast, investigation into genetic markers has not indicated that genetic variants or combinations of them could help to identify predictors of BP response after RDN [43]. To date, the only clinical variable consistently associated with BP response to date has been the severity of baseline BP [44], but this is probably just an example of Wilder’s principle of initial values [45].

Furthermore, it is critical to differentiate the concept of a “procedure responder” from a “*treatment responder*” in the context of RDN. A *procedure responder* refers to a patient in whom the renal denervation procedure successfully disrupts the renal sympathetic nerves, as confirmed by physiological, imaging, or biological markers indicative of effective nerve ablation. In contrast, a *treatment responder* is a patient who demonstrates meaningful clinical improvement (in hypertension, commonly accepted as reduction in blood pressure) following the RDN procedure. Ideally, treatment responders should be a subset of procedure responders; however, this is not always the case due to interindividual variability and procedure limitations. In preclinical models, tools such as norepinephrine spillover technology, high throughout tandem mass spectrometry, and histological staining have been validated to assess the efficacy of renal denervation, though these methods have limited value in clinical practice [46].

To bridge this gap, several approaches have emerged. For example, Symap Medical group (Suzhou, China) developed a mapping and selective renal denervation (msRDN) system. This technique involves intra-arterial electrical stimulation to identify “hot spots”—sites where stimulation elicits a systolic blood pressure rise of more than 5 mmHg. These regions are selectively ablated, while non-responsive (“cold”) areas are left intact. Post-ablation stimulation confirms successful nerve disruption. On average, four hot spots are ablated per renal artery, and clinical studies have reported a significant reduction in the drug index (calculated as the number of antihypertensive drug classes multiplied by the sum of their doses), with a favorable safety profile [47]. Another emerging method involves transvascular pacing of the aortorenal ganglia, which typically induces renal artery spasm—a response absent after effective denervation in a preclinical pig model [48].

**Figure 1:**
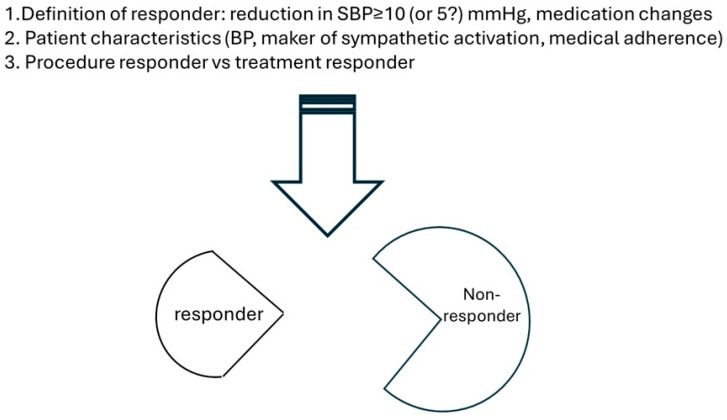
Key Steps in Identifying Responders to RDN.

Ultimately, a team approach involving primary care providers, hypertension specialists, proceduralists, and clinical researchers is critical to determine the ideal profile(s) for RDN (Figure 1). Such collaboration will be key to integrating this therapy into contemporary hypertension management.

The utmost goal of blood pressure treatment is to reduce the risk of cardiovascular events. While MACEs (Major Adverse Cardiovascular Events) are commonly used as primary outcomes in cardiovascular trials, their application in antihypertensive trials presents several challenges. Firstly, a MACE is a heterogenous composite end point, which can lead to markedly different interpretations and conclusions, especially when combining procedure safety and treatment efficacy [49]. Secondly, considering the modest BP-lowering effect of RDN, the sample size required to adequately power a trial using MACE as the primary outcome would exceed 20,000 patients—rendering such a study impractical due to costs and the need for prolonged follow up in a sham-controlled procedure trial [50]. Consequently, BP reduction remains an acceptable surrogate for cardiovascular outcome data in RDN studies.

Moreover, a key advantage of the BP-lowering effects of RDN over pharmacologic therapies is its continuous, “always on” effect, providing 24 h BP control without the variability of medication adherence. Identifying the most meaning BP metrics, beyond the standard 24 h ambulatory BP monitoring, is therefore essential to assess the full clinical benefit of RDN. Of particular interest are nocturnal and early morning BP values, which are increasingly recognized as critical indicators of cardiovascular risk. Morning hours are generally considered a higher-risk period for myocardial infarction, stroke, and sudden death, partly due to BP surges that may trigger the rupture of atherosclerotic plaques or vascular events [51]. Similarly, nocturnal hypertension—defined as nighttime BP ≥ 120/70 mmHg—is an independent predictor of cardiovascular events and a target for treatment per the latest ESC/ESH guidelines [52]. The coexistence of nocturnal hypertension and morning surge poses a “tsunami” of risk for strokes and other cardiovascular events [53].

Data from both clinical trials and registry analyses have confirmed that RDN significantly reduces nighttime and early morning BP, whereas the sham control (medication only) group showed minimal or no changes from baseline [54,55]. These intriguing findings suggest that nighttime and early morning BP readings may be the most sensitive and clinically relevant metrics for assessing RDN efficacy.

One major limitation, however, is the discomfort and interruption of sleep from repeated cuff inflation, particularly when using upper arm-based devices. While the AHA currently does not recommend wrist cuff-based devices, the ESC/ESH guidelines acknowledge them as a possible alternative. Encouragingly, new oscillometric wrist cuff devices are under development, offering improved comfort and reduced muscle pressure than the traditional upper-arm cuff [56]. Coupled with the recent advancement in digital healthcare technologies, there is great potential to more accurately track RDN outcomes and enhance patient care in contemporary clinical practice.

Beyond BP reduction, there is growing interest in the potential benefits of RDN on metabolic health, sympathetic overactivity-related disorders, and overall cardiovascular risk reduction. Some studies suggest that RDN may improve insulin sensitivity, reduce left ventricular hypertrophy, diastolic dysfunction, and new onset/recurrence of atrial fibrillation and positively influence kidney function, making it a multifaceted intervention beyond just hypertension control [57]. By using a novel modeling approach of Global SYMPLICITY Registry (GSR) data (*n* = 2651), Schmieder et al. demonstrated that significant absolute reductions in major adverse cardiovascular events over 3 years compared with the projected control (8.6 +/− 0.7% actual vs. 11.7 +/− 0.9% projected control; *p* < 0.01) [58]. Indeed, a randomized sham-controlled prospective outcome trial on RDN is urgently needed to provide definitive evidence regarding the overall benefit of RDN and further guide the integration of RDN into clinical practice.

The primary goal of hypertension treatment should aim at both improving life prognosis as well as the quality of life. However, the awareness of hypertension and the initiation of antihypertensive medications can sometimes negatively impact the quality of life due to medication side effects and the need for lifestyle modifications. This is particularly relevant as high blood pressure itself often has no direct impact on a patient’s subjective symptoms, making treatment adherence challenging. The “stay-on” antihypertensive effect of RDN presents a promising alternative by potentially reducing or even eliminating the need for long-term antihypertensive medications [59]. This could significantly improve patients’ quality of life and ultimately have health economic benefits for society as hypertension affects over half of the adults. A recent study by Kandzari et al. estimated that RDN could add 0.34 quality-adjusted life years (QALY) at a cost of USD 11,275, resulting in an incremental cost-effectiveness ratio (ICER) of USD 32,732 per QALY [60].

## 6. Perspective

The FDA’s approval of RDN represents a major milestone in hypertension management, providing a novel treatment option for patients with resistant hypertension. According to Watson et al., using data from the National Health and Nutrition Examination Survey (NHANES) (2009–2020), an estimated 21.5% (95% CI, 20.7–22.3%) of U.S. adults with hypertension would qualify for RDN under FDA indications [61]. This translates into a substantial high-risk population, vulnerable to cardiovascular events, stroke, and end-organ damage, emphasizing the urgent need for effective therapeutic strategies.

However, maximizing the benefits of RDN will require the following:

- Careful patient selection to identify those who will derive the most benefit;
- A dedicated multidisciplinary approach to ensure comprehensive management;
- Ongoing research to refine procedural techniques and further define the ideal patient profile.

As clinical trials and real-world studies continue to provide deeper insights, integrating RDN with pharmacotherapy and lifestyle modifications has the potential to redefine hypertension management, offering a personalized and holistic approach for patients with resistant or difficult-to-treat hypertension.

## Figures and Tables

**Table 1:** Characteristics of major RDN catheter systems.

Technology and Catheter	Design	Access Site and Size	Ablation Sites	Status
**Radiofrequency**				
Symplicity Spyral Catheter (Medtronic, Minneapolis, MN, USA)	Multiple electrode (4 monopolar electrode), helical design, 60 s per ablation cycle	F (6Fr)	Main and accessory arteries, including branches (same catheter diameter 3–8 mm)	FDA approval 2023
Netrod (Shanghai Golden Leaf MedTec Co., Ltd., Shanghai, China)	Multiple electrode (6 monopolar electrode), basket-shaped tip, 120 s per cycle	F (8Fr)	Main and accessible renal arterial vessels (same catheter diameter 3–12 mm)	Sham-controlled trial (EHJ 2024)
Iberis (Angiocare and Terumo, Somerset, NJ, USA)	Multielectrode (4 monopolar electrode), helical design, 60 s per ablation cycle	F/R (6Fr)	Main and accessory arteries, including branches (same catheter diameter 3–8 mm)	Iberis-HTN trial, sham controlled, blinded (Circulation 2024)
**Ultrasound**				
Paradise (RECOR, Palo Alto, CA, USA)	Piezoelectric ceramic transducer within a fluid-cooled, low-pressure balloon, 7 s per emission	F (7Fr)	Main and accessory arteries, including branches (different catheters for diameter 3–8 mm)	FDA approval 2023
TIVUS (SoniVie, Rehovot, Israel)	Nonocclusive, free floating in the lumen, high ablation depth	R (4Fr)	One size fits all catheter	REDUCED-1 Pilot study (TCT 2024) THRIVE trial (ongoing)
**Neurolysis**				
Peregrine (Ablative Solutions, Wakefield, MA, USA)	Infusion catheter with three extendable ultrathin microneedles into perivascular space of renal arteries (0.6 mL dehydrated alcohol per treatment)	F (7Fr)	Main and accessory arteries (supply >20% renal parenchyma, maximum of one accessory artery treated per side), including branches (diameter 3–7 mm)	Target BP trial (Circulation 2024)

F: femoral access; R: radial access; Fr: French.

**Table 2:** A brief list of important randomized clinical trials of RDN.

Study	Year of Pub	# of Patients	Patient Characteristics	Device	Medications	Primary Endpoint	Additional Data
Symplicity HTN-3	2014	RDN = 364 Sham = 171	RH (5.1 vs. 5.2 meds in RDN and sham)	Symplicity Flex catheter (radiofrequency)	Protocol called for stable background of medications, but 39% of patients changed medications during study.	Mean change in office SBP at 6 months was −14 ± 24 in the RDN group vs. −12 ± 26 in the sham group (*p* = 0.26)	3-year follow-up showed a further reduction in office SBP of 26.4 ± 25.9 in RDN vs. 5.7 ± 24.4 in the sham (*p* ≤ 0.0001).
RADIANCE-HTN SOLO	2018	RDN = 74 Sham = 72	Ambulatory BP ≥ 135/85 and <170/105 after a 4-week d/c of up to 2 meds	Paradise (ultrasound)	No background antihypertensive medications	Reduction in daytime ambulatory SBP with RDN (−8.5 ± 9.3), Sham (−2.2 ± 10.0); *p* = 0.0001	For 51 patients in the RDN group with 36-month follow-up, office BP decreased by 18/11 ± 15/9 from baseline (*p* < 0.001 for both).
SPYRAL OFF MEDS	2020	RDN = 166 Sham = 165	150 ≤ Office SBP < 180, and office DBP ≥ 90, and 140 ≤ mean 24 h SBP < 170	Spyral catheter (radiofrequency)	No background antihypertensive medications	Reduction in 24 h SBP at 3 months was −4.7 [−6.4, −2.9] in RDN and −0.6 [−2.1, 0.9] in sham	Patients meeting ‘escape criteria’ (office SBP ≥ 180) were lower in the RDN (9.6%) than in the sham (17.0%) within 3 months (*p* = 0.032).
RADIANCE-HTN TRIO	2021	RDN = 69 Sham = 67	Office BP ≥ 140/90 while on at least 3 medications of different classes including a diuretic for at least 4 wks	Paradise (ultrasound)	Stable dose of a triple medication pill (amlodipine, valsartan and HCTZ)	Reduction in daytime ambulatory SBP at 2 months in RDN (−8.0 [IQR –16.4 to 0.0]) vs. Sham (–3.0 [–10.3 to 1.8]) adjusted *p* = 0.022.	Similar 6-month daytime ambulatory BP measurements in both groups (138.3 ± 15.1 with uRDN vs. 139.0 ± 14.3 in the sham group although fewer medications were added in the RDN group (0.7 ± 1.0 vs. 1.1 ± 1.1 medications; *p* = 0.045)
RADIANCE II	2023	RDN = 150 Sham = 74	140/90 ≤ Office BP ≤180/120 mm Hg while at least 4 wks on 0–2 medications of different classes	Paradise (ultrasound)	No background antihypertensive medications	Mean reduction in daytime ambulatory SBP at 2 months was −7.9 ± 11.6 with RDN vs. −1.8 ± 9.5 (*p* < 0.001)	Controlled daytime ambulatory BP was achieved in 18.8% of RDN group and 4.8% in the sham procedure group
SPYRAL ON MEDS (expansion)	2023	RDN = 206 Sham = 131	Uncontrolled HTN (2.2 vs. 2.3 meds in RDN and Sham)	Spyral catheter (radiofrequency)	Stable dose of 1–3 antihypertensive medications	Reduction in mean 24 h ambulatory SBP at 6 months RDN (−6.5 ± 10.7) vs. sham (−4.5 ± 10.3); *p* = 0.12.	Office SBP (−9.9 ± 13.9 vs. −5.1 ± 13.2; *p* = 0.0015) and DBP (−5.2 ± 8.8 vs. −3.3 ± 8.2; *p* = 0.041) were lower in RDN at 6 months
TARGET BP 1	2024	RDN = 148 Sham = 153	150/90 ≤ Office BP ≤180/90, and 135 ≤ mean 24 h SBP ≤170 mm Hg despite on 2 to 5 medications	Peregrine alcohol-mediated RDN	Stable dose of antihypertensive medications	24 h ambulatory SBP at 3 months was −10.0 ± 14.2 in the RDN and −6.8 ± 12.1 in the sham; *p* = 0.0487.	No significant differences in office SBP or DBP between the RDN and sham control groups. At 30 days, major adverse event rate was 4.7% for the RDN group and none for the sham control group (*p* = 0.007)

RDN = renal denervation; RH = resistant hypertension; SBP = systolic blood pressure; DBP = diastolic blood pressure; uRDN = ultrasound RDN.

**Table 3:** Summary of recommendations for RDN from major societies and countries.

Society	Year	Role as Adjunctive Therapy	Indications						Contra- Indications/Uncertainties
			Resistant HTN	Uncontrolled HTN Due to Intolerance	Uncontrolled HTN Due to Nonadherence	ABPM Confirmation	R/O 2nd Causes	Favor Patients with High Cardiovascular Risks	
SCAI	2021, 2023	Y	+	+	+	N/A	+	+	NS *
AHA	2024	Y	+	+	+	N/A	+	N/A	Y **
ESH ***	2023	Y	+	+	+	+	+	N/A	NS
ESC	2023	Y	+	+	+	+	+	+	NS ^#^
Netherland ^##^	2022	Y	+	+	+	+	+	N/A	NS
Britain /Ireland ^###^	2019, 2023	N	+					N/A	NS
Taiwan	2022	Y	+	+	+	N/A	+	+ ^@^	NS
Japan	2024	Y	+	+	+	N/A	+	N/A	Y ^@@^
Malasia ^@@@^	2022	Y	+	+	+	N/A	+	+	NS

Y = yes; N/A= not applicable; NS = Not specified. *, mentioned to be cautions about patients with “…low GFR, single functioning kidney, atrophic kidney, renal tumor, renal artery aneurysm, renal stent, renal transplantation, significant renal artery stenosis…”. **, Contraindicated in patient with “…Pregnancy, fibromuscular dysplasia, stented renal artery, renal artery aneurysm, known kidney or secreting adrenal tumors, single renal artery stenosis”; uncertain about effects in patients with “…Stage 1 hypertension, isolated systolic hypertension, Stage 4 and 5 CKD, single kidney, kidney transplant recipients (on native nonfunctional kidneys) …”. ***, recommend in patients with “…eGFR > 40 mL/min/1.73 m^2^…”, recommend to “… only perform in experienced specialized centers…”. # Uncertain about effects in patients with “…Isolated systolic hypertension, kidney transplant receipts, stage 4,5 CKD, FMD, untreated secondary hypertension, single functioning kidney, hemodialysis…”. ## To qualify for resistant hypertension, patients need to be on three antihypertensive medications, at least one diuretic for at least three months. To qualify for intolerance, patient needs to have documented intolerance to at least three different classes of antihypertensive drugs. ###, Only be used with special arrangements for clinical governance, consent and audit or research. Evidence of medication concordance by urinary analysis or direct observation. @, Also features indicative of neurogenic hypertension. @@, Contraindicated in patients with “…renal aneurysm, renal artery stenosis or unsuitable renal artery anatomy, eGFR < 30 mL/min/1.73 m^2^…”. To qualify for uncontrolled hypertension, office BP (≥140/90 mmHg) and/or out-of-office BP (24 h ambulatory BP ≥ 130/80 mmHg, daytime ambulatory BP ≥ 135/85 mmHg, nighttime ambulatory BP ≥ 120/70 mmHg, morning/evening home BP ≥ 135/85 mmHg, or nighttime home BP ≥ 120/70 mmHg (despite adequate lifestyle modification and treatment with maximally tolerated dosages of three or more antihypertensive medications including a diuretics). @@@, RDN should be considered early to prevent target organ damage.
